# Molecular Epidemiology of Imported Cases of Leishmaniasis in Australia from 2008 to 2014

**DOI:** 10.1371/journal.pone.0119212

**Published:** 2015-03-03

**Authors:** Tamalee Roberts, Joel Barratt, Indy Sandaradura, Rogan Lee, John Harkness, Deborah Marriott, John Ellis, Damien Stark

**Affiliations:** 1 Department of Microbiology, SydPath, St. Vincent’s Hospital, Victoria St, Darlinghurst, N.S.W, Australia; 2 School of Medical and Molecular Sciences, University of Technology, Sydney, Ultimo, N.S.W, Australia; 3 i3 Institute, University of Technology, Sydney, Ultimo, N.S.W, Australia; 4 Centre for Infectious Diseases and Microbiology Laboratory Services, ICPMR, Westmead Hospital, Westmead, N.S.W, Australia; Pasteur Institute of Iran, IRAN, ISLAMIC REPUBLIC OF

## Abstract

Leishmaniasis is a vector borne disease caused by protozoa of the genus *Leishmania*. Human leishmaniasis is not endemic in Australia though imported cases are regularly encountered. This study aimed to provide an update on the molecular epidemiology of imported leishmaniasis in Australia. Of a total of 206 biopsies and bone marrow specimens submitted to St Vincent’s Hospital Sydney for leishmaniasis diagnosis by PCR, 55 were found to be positive for *Leishmania* DNA. All PCR products were subjected to restriction fragment length polymorphism analysis for identification of the causative species. Five *Leishmania* species/species complexes were identified with *Leishmania tropica* being the most common (30/55). Travel or prior residence in a *Leishmania* endemic region was the most common route of acquisition with ~47% of patients having lived in or travelled to Afghanistan. Cutaneous leishmaniasis was the most common manifestation (94%) with only 3 cases of visceral leishmaniasis and no cases of mucocutaneous leishmaniasis encountered. This report indicates that imported leishmaniasis is becoming increasingly common in Australia due to an increase in global travel and immigration. As such, Australian clinicians must be made aware of this trend and consider leishmaniasis in patients with suspicious symptoms and a history of travel in endemic areas. This study also discusses the recent identification of a unique *Leishmania* species found in native kangaroos and a potential vector host which could create the opportunity for the establishment of a local transmission cycle within humans.

## Introduction

Leishmaniasis is a vector-borne disease caused by protozoa of the genus *Leishmania*. The disease is transmitted via sand flies of the genus *Phlebotomus* in the old world (Europe, Asia and Africa) and *Lutzomyia* in the new world (the Americas) [1]. There are over 14 species of *Leishmania* which may cause up to three different clinical syndromes [2,3]: cutaneous leishmaniasis (CL) manifests as ulcerated skin lesions; mucocutaneous leishmaniasis (MCL) affects the mucous membranes of the nose, mouth and throat and can lead to partial or total destruction of the associated membranes; and visceral leishmaniasis (VL) is a systemic, potentially lethal disease caused by parasites of the *Leishmania donovanii* complex [4,5]. There are an estimated 1.2 million cases of CL and 400,000 cases of VL reported annually worldwide [6], while MCL is a much rarer illness. In Australia leishmaniasis is an imported disease with no locally acquired human infections described to date. Imported cases of leishmaniasis are becoming increasingly common in non-endemic regions such as Australia, North America and Northern Europe, due to increased international travel, immigration and deployment of defence personnel to endemic areas [5,7,8]. There are several clinically important *Leishmania* sp. and each are morphologically indistinguishable. Whilst a patient’s travel history may facilitate determination of the causative species, different species often occupy overlapping geographic ranges [5]. As the response to treatment in clinical cases of leishmaniasis may be species specific [9], accurate speciation is important to determine optimal treatment and precise prognosis.

The life cycle of *Leishmania* sp. is a two-stage cycle involving a vertebrate host and an insect vector. During this cycle, the parasite exists in two different morphological states: as amastigotes inside phagocytes of their vertebrate hosts, or as flagellated promastigotes within the gut of their insect vector, usually a phlebotamine sand fly [10]. Within its endemic range, leishmaniasis is a common zoonoses and infects a variety of animals including feral dogs, marsupials, rodents and domestic animals [3,11]. Until recently Australia was thought to be free of leishmaniasis though with the confirmation of CL in native Australian macropods, Antarctica is now the only continent thought to be *Leishmania* free [12,13]. The discovery of a native species of *Leishmania* in Australia raises two important queries; (1) whether this native *Leishmania* sp. has the capacity to cause human disease under certain circumstances, and (2) whether the proposed native Australian insect vector has the capacity to transmit other, clinically important *Leishmania* sp.

Over the last decade the number of cases of imported leishmaniasis has doubled in the Netherlands and tripled in the UK [14].There are few recent reports describing imported cases of leishmaniasis in Australia [15–20] and as a consequence, current data on the molecular epidemiology of Australian imported leishmaniasis is lacking. Furthermore, with the increase in global travel, immigration to Australia, and the despatch of Australian military personnel to endemic regions, it is important that the current status of leishmaniasis in Australia is regularly monitored. Therefore, the aim of this study was to provide a current, large-scale report on the molecular epidemiology of imported leishmaniasis in Australia.

## Materials and Methods

A total of 206 punch biopsies or bone marrow aspirates from patients with suspected leishmaniasis infection were submitted to the Department of Microbiology, St. Vincent’s Hospital, Sydney, Australia between July 2008 and March 2014. DNA was extracted from all tissue samples using a Qiagen Tissue extraction kit (Qiagen, Hilden, Germany) and the Qiagen Biorobot EZ1. The DNA extracts were used as template for a conventional PCR assay targeting the ITS1 region of *Leishmania* sp., which has been previously described [21]. A portion of all PCR products was subjected to agarose gel electrophoresis on a 2% agarose gel (Life Technologies). Gels were visualised under UV light to confirm the presence of a PCR product from 300–350bp in size, which is indicative of a *Leishmania* positive sample. Confirmation of the causative *Leishmania* species was carried out on all PCR positive samples by restriction fragment length polymorphism (RFLP) analysis. For RFLP analysis, PCR products were digested using the restriction enzyme *Hae*III (as per [7]). Digestion was carried out for 60 minutes using the conditions recommended by the supplier (Sigma-Aldrich). Restriction fragments were then subjected to agarose gel electrophoresis on a 4% gel (Life Technologies) and viewed under UV light for visualisation of the restriction patterns. Determination of the causative *Leishmania* species was performed by comparing the resulting restriction patterns to those previously published [7]. All PCR-RFLPs were accompanied by a positive control consisting of *Leishmania* DNA extracted from a clinical isolate of *Leishmania tropica* which had been previously isolated into culture in the Department of Microbiology lab at St Vincent’s Hospital, Sydney.

### Ethics Statement

This retrospective study was approved by the institutional ethics review committee at St. Vincent’s Hospital, Sydney (HREC reference: LNR /14/SVH/374, SSA reference: LNRSSA/14/SVH/378) and all patient details were de-identified.

## Results

**Fig 1:**
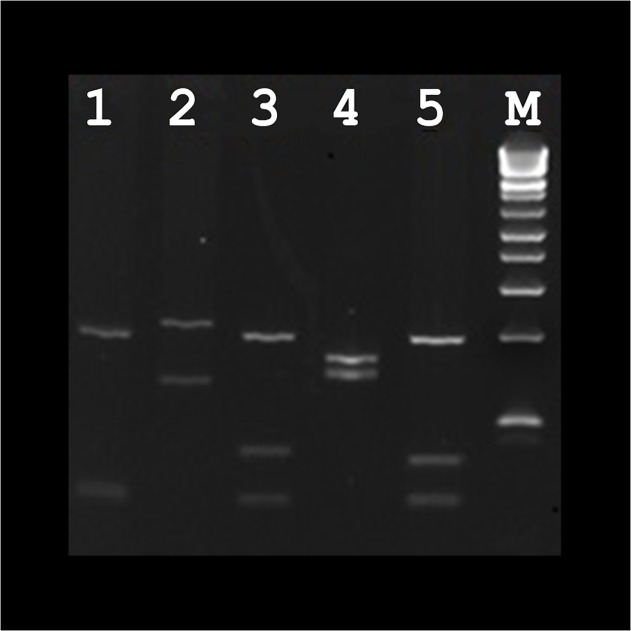
Digestion of amplified ITS1 regions with the restriction endonuclease *Hae*III of different species of *Leishmania* from isolates from this study on a 4% agarose gel. Lane 1: *L*. *tropica*, lane 2: *L*. *major*, lane 3: *L*. *donovani* complex, lane 4: *L*. *braziliensis* complex, lane 5: *L*. *mexicana*. A 100bp ladder was used as the molecular size marker (M).

Of the 206 samples submitted to the Department of Microbiology between July 2008 and March 2014 for investigation of leishmaniasis, *Leishmania* infection was confirmed in 55 patients by PCR. The vast majority of PCR confirmed cases were male (n = 41) with only 14 confirmed female cases. CL was the most common clinical manifestation (n = 52, 94%) with VL in three patients (5%). Travel to *Leishmania* endemic regions was the source of infection in most cases. Patients consisted of 5 defence force personnel who had toured in an endemic country as part of their duties, 28 people who had travelled overseas for holiday or to visit family in an endemic area and 21 patients who had immigrated to Australia from an endemic country. For two patients who had a history of travel or who had immigrated to Australia, the exact region in which they had lived or travelled was not specified. A history of travel or residence in Afghanistan was noted in the majority of cases (n = 24) while 13 patients reported travel or residence in other Middle Eastern countries. There were nine patients that had reportedly travelled in Central or South America and four patients who had travelled to countries in the Mediterranean, two of whom acquired their infection from Southern Spain. Five species of *Leishmania*/*Leishmania* complex organisms were identified; 30 *L*. *tropica*, 7 *L*. *donovanii* complex, 5 *L*. *braziliensis* complex, 6 *L*. *major* and 2 *L*. *mexicana*. For five specimens, speciation could not be determined due to the presence of a very weak positive PCR product resulting in a restriction pattern that was difficult to interpret. Most patients acquired leishmaniasis in the Old World (n = 43). For patients with CL, ulcerative lesions on the legs (n = 14) and arms (n = 12) were the most common manifestations. One patient who was affected by VL acquired their infection by transplacental transmission. The patient’s mother was a Sudanese refugee who had been living in Australia for two years prior to becoming pregnant. This patient died from infection at 2 years old. No cases of MCL were identified in this study. A summary of these results can be found in Table 1. Fig. 1 shows the digestion of amplified ITS1 regions with the restriction endonuclease *Hae*III of different species of *Leishmania* from isolates from this study on a 4% agarose gel.

**Table 1:** Patient details, associated risk factor, clinical presentation (site if known) and species identified.

Patient	Age	Sex	Risk Factor	Country	Clinical presentation	*Leishmania* species (identified by RFLP)
1	67	M	Travel	Peru	CL (plaques on chest)	*L*. *mexicana*
2	39	M	Travel	Mexico	CL (elbow)	*L*. *mexicana*
3	28	F	Travel	Costa Rica	CL	*L*. *braziliensis* complex
4	61	F	Travel	Peru	CL (cheek)	*L*. *braziliensis* complex
5	30	F	Travel	Colombia	CL	*L*. *braziliensis* complex
6	29	F	Travel	French Guyana	CL (elbow)	*L*. *braziliensis* complex
7	52	M	Travel	Colombia	CL (calf)	*L*. *braziliensis* complex
8	2	M	Trans placental	Australia (Sudan)	VL	*L*. *donovanii* complex
9	69	F	Travel	Asia, Africa, South America	VL	*L*. *donovanii* complex
10	48	M	Travel	Southern Spain	VL	*L*. *donovanii* complex
11	60	M	Travel	Malta	CL (buttock)	*L*. *donovanii* complex
12	54	M	Travel	Asia, Africa, South America	CL (leg)	*L*. *donovanii complex*
13	40	M	Travel	Southern Spain	CL (calf)	*L*. *donovanii* complex
14	76	M	Immigrant	Italy (lived in Australia for 30 yrs)	CL	*L*. *donovanii* complex
15	42	M	Immigrant	Middle East	CL (elbow)	*L*. *major*
16	24	M	Immigrant	Afghanistan	CL (foot)	*L*. *major*
17	36	M	Travel	Afghanistan	CL (arm)	*L*. *major*
18	31	M	Army	Iraq	CL	*L*. *major*
19	21	M	Army	Iraq	CL	*L*. *major*
20	31	M	Army	Syria	CL (back)	*L*. *major*
21	33	M	Army	Afghanistan	CL	*L*. *tropica*
22	23	M	Army	Afghanistan	CL	*L*. *tropica*
23	18	M	Travel	Syria, Iraq	CL (arm)	*L*. *tropica*
24	36	M	Travel	Middle East	CL	*L*. *tropica*
25	43	F	Travel	Middle East	CL (elbow)	*L*. *tropica*
26	5	M	Travel	Middle East	CL (foot)	*L*. *tropica*
27	49	F	Travel	Middle East	CL (foot)	*L*. *tropica*
28	5	M	Travel	Middle East	CL	*L*. *tropica*
29	22	M	Travel	Middle East	CL	*L*. *tropica*
30	66	F	Travel	Iran	CL (face)	*L*. *tropica*
31	8	F	Travel	Afghanistan	CL	*L*. *tropica*
32	26	F	Travel	Afghanistan	CL	*L*. *tropica*
33	12	M	Travel	Afghanistan	CL (cheek)	*L*. *tropica*
34	20	M	Immigrant	Afghanistan	CL	*L*. *tropica*
35	23	M	Immigrant	Afghanistan	CL	*L*. *tropica*
36	69	F	Immigrant	Afghanistan	CL	*L*. *tropica*
37	2	M	Immigrant	Afghanistan	CL	*L*. *tropica*
38	2	M	Immigrant	Afghanistan	CL (cheek)	*L*. *tropica*
39	45	M	Immigrant	Afghanistan	CL (hand)	*L*. *tropica*
40	26	M	Immigrant	Afghanistan	CL (arm)	*L*. *tropica*
41	16	M	Immigrant	Afghanistan	CL	*L*. *tropica*
42	27	M	Immigrant	Afghanistan	CL (leg)	*L*. *tropica*
43	18	M	Immigrant	Afghanistan	CL (ankle)	*L*. *tropica*
44	30	M	Immigrant	Afghanistan	CL (thumb)	*L*. *tropica*
45	30	M	Immigrant	Afghanistan	CL (finger)	*L*. *tropica*
46	36	M	Immigrant	Afghanistan	CL (groin)	*L*. *tropica*
47	16	M	Immigrant	Afghanistan	CL (ear)	*L*. *tropica*
48	36	F	Immigrant	Afghanistan	CL (arm)	*L*. *tropica*
49	18	M	Immigrant	Afghanistan	CL (arm)	*L*. *tropica*
50	1	M	Immigrant	Afghanistan	CL (ankle)	*L*. *tropica*
51	28	M	Travel	Pakistan	CL (nose)	No ID*
52	36	F	Travel	Panama	CL	No ID*
53	26	F	Travel	Peru	CL (arm)	No ID*
54	34	M	Travel	-	CL (calf)	No ID*
55	26	M	Immigrant	-	CL	No ID*

*Due to a very weak PCR positive result which made restriction patterns difficult to interpret

## Discussion

Whilst previous cases of imported leishmaniasis have been described, this is the largest series to examine the molecular epidemiology of imported leishmaniasis in Australia. One previous Australian study describes a case in a patient who acquired leishmaniasis during travel to Belize [19]. Another describes an imported case involving an 18 year old male who developed VL after a trip to Greece [18]. A third report describes four Afghani refugees who were diagnosed with CL following entry into Australia [20]. The second largest Australian study to date described 20 cases of imported leishmaniasis diagnosed over a three year period [16]. The present study provides the most recent update and reports a further 55 cases of leishmaniasis diagnosed over a six year period. This study also provides information on the causative species based on the results of a PCR-RFLP technique previously described [7,21]. *Leishmania tropica* was the most common species identified in this study (55%) which is higher than the previous Australian study which reported *L*. *tropica* in only 35% of cases [16]. The slight increase in imported *L*. *tropica* infections in comparison to the previous study is probably attributable to the recent increase in immigration from Afghanistan to Australia and the deployment of defence personnel to Afghanistan. In the previous Australian study [16] members of the *L*. *braziliensis* complex were the second most common cause of leishmaniasis, in contrast to the present study in which *L*. *donovanii* complex cases were the second most common (n = 7). The proportion of imported *L*. *major* and *L*. *mexicana* cases reported in this study is similar to those reported in the previous Australian study [16].

In the present study, CL was the most common clinical syndrome described (n = 52). Ulcers on the arms and legs were the most common manifestation in CL patients. Consequently, cutaneous lesions on the limbs of travellers returning from endemic *Leishmania* regions should immediately alert clinicians to the possibility of *Leishmania* infection. Unsurprisingly, no cases of MCL were observed in this study, which is in agreement with previous reports of imported leishmaniasis. Whilst MCL is the most disfiguring of the three *Leishmania* syndromes, it is also the least common [3]. MCL usually occurs as a result of infections with New World *Leishmania* species (usually *Leishmania braziliensis* complex) and generally occurs concurrently with or following a cutaneous infection, albeit rarely [3].

*Leishmania tropica* was the most common species identified in the study cohort which coincides with the patients' travel history where more than half of the patients had a history of travel to or immigration from Afghanistan or the Middle East. A report from the Netherlands described *L*. *major* as the most common species identified in a cohort consisting predominantly of Dutch soldiers who had been deployed to Afghanistan [5]. In our study it was observed that three of the five defence personnel were also infected with *L*. *major*. In addition, in the Dutch study *L*. *tropica* was only identified in civilians that had a history of travel or residence in Afghanistan [5]. Most of the cohort examined in this study were infected with *L*. *tropica* and it should be noted that the vast majority were civilian immigrants from Afghanistan. *L*. *major* is commonly found in rural areas whereas *L*. *tropica* is predominantly found in urban areas. This could explain the difference in species found in people who had travelled from the same country as defence personnel are more likely to be deployed to rural areas and therefore more likely to be infected with *L*. *major*.

In total we were able to identify 25 other series [5,7,16,22–41] reporting three or more cases of imported leishmaniasis which also included demographic and travel history together with speciation of isolates, summarised in S1 Table (S1 Published series of travel related leishmaniasis). Similar to our study most imported cases tend to occur in men, presumably due to risk taking behaviour, and present as the cutaneous form of the disease. In contrast, New World species were much less common in our study and the Old World species were mostly acquired in the Middle East rather than Southern Europe. In a study from the UK, the majority of Old World leishmaniasis cases were reportedly acquired whilst travelling in Southern Europe [41]. There were only three patients in the present study who acquired their infection after travel to a Southern European country (Malta and Spain) and a fourth patient from this study is assumed to have acquired their infection in Southern Europe as they had immigrated to Australia from Italy 30 years prior. These differences are likely explained by the differences in travel patterns of Australian residents and the large number of immigrants in the study. Deployment of Australian troops to the Middle East during this period likely contributed as well.

A recent report by Alvar et al. describes 98 countries endemic for leishmaniasis from five continents [6]. Of these, Afghanistan, Colombia, Syria, Peru and Sudan were among the top 10 countries described as having the highest incidence of CL. Not surprisingly, 33 of the 55 patients from this study acquired their infection in one of these five countries. Due to increased global travel, immigration and deployment of defence personnel to *Leishmania* endemic areas there is an increased need for clinicians in non-endemic areas to be more aware of leishmaniasis and to consider it in patients displaying clinical manifestations resembling those of CL, VL or MCL. Furthermore, clinicians should also be aware that in rare cases, paediatric VL can occur in patients with no history of travel. In these unusual cases, the familial history should also be considered given the potential for transplacental transmission of *Leishmania* to occur as observed once in this study.

Accurate speciation in clinical cases of leishmaniasis is not only important from an epidemiological perspective, but can be important for predicting the clinical outcome and selecting an appropriate treatment regimen. New World leishmaniasis caused by species within the *L*. *braziliensis* complex (*L*. *braziliensis*, *L*. *guyanensis*, *L*. *panamensis* and *L*. *peruviana*) are more likely to lead to secondary MCL than other New World species. As MCL can occur up to two years after cutaneous lesions resolve [42], knowledge of the causative species can facilitate diagnosis should lesions appear on the mucous membranes at a later time. Furthermore, *L*. *braziliensis* complex organisms are resistant to miltefosine while other species are not [14], so miltefosine is unlikely to be effective in such cases.

PCR is currently the tool of choice for diagnosis of leishmaniasis. Traditionally microscopy, histopathology and culture were used though these techniques do not differentiate between *Leishmania* species. Prior to the advent of PCR, isoenzyme analysis was the gold standard for speciation of *Leishmania* sp. though this technique is comparatively laborious and requires prior cultivation of parasites *in vitro* [7]. Several PCR-based tools have been developed which are capable of differentiating between certain species and/or complexes of *Leishmania*, though each of these has its advantages and limitations [7,43–46]. One study showed that Kinetoplast DNA (kDNA) had the highest sensitivity for the detection of *Leishmania* sp. over ITS1 PCR and splice leader mini-exon PCR, however this technique does not allow for speciation [47]. RT-PCRs have been developed but most have the limitation of only differentiating to complex level and not having the ability to speciate within that complex. The advantage of this though is that RT-PCR is much faster than conventional PCR, which has to be followed by either RFLP or sequencing for any kind of speciation, if only the complex level is desired. Generally PCR is highly sensitive and when coupled with RFLP analysis, the assay employed in this study, can differentiate between most *Leishmania* species. This PCR-RFLP does have its limitations however, as it cannot differentiate between species within the *L*. *donovani* complex (*L*. *donovani* and *L*. *infantum*/*L*. *chagasi*) and those within the *L*. *braziliensis* complex. Sequencing of PCR products to differentiate between species is also complicated by the heterogenous nature of *Leishmania* ITS1 sequences. The ribosomal RNA (rRNA) genes exist in eukaryotic genomes as tandem repeats with many copies [48,49]. In some protozoa (e.g. *Toxoplasma gondii* and relatives), the ITS1 region is identical for each copy [50,51], which is conducive to sequencing. In contrast, the ITS1 region in individual *Leishmania* isolates varies greatly between copies [52]. As a result, PCR products derived from the ITS1 of *Leishmania* do not produce a clean sequencing read. Regardless, because the rRNA genes exist in multiple copies in the genome, there is still the benefit of increased sensitivity for PCRs targeting these genes compared to those targeting single copy genes.

There have been no confirmed cases of locally acquired human leishmaniasis in Australia to date. However, a recent report describes naturally acquired cases of CL in Australian native macropods including the red kangaroo, the black wallaroo and the agile wallaby [53]. A genetic characterisation of the causative *Leishmania* species suggests it is a unique species that is probably endemic to Australia. While *Leishmania* DNA has never been detected in Australian species of the phlebotamine sand-fly, these Australian *Leishmania* parasites were recently detected in a previously undescribed species of day-feeding midge from the subgenus *Forcipomyia* (*Lasiohelea)* [10]. While there is little evidence to suggest that this macropod-infecting *Leishmania* sp. can infect humans, the possibility that the Australian midge vector could transmit pathogenic *Leishmania* sp. is a cause for concern. In a recent study, midges of the genus *Culicoides* (family Ceratopogonidae) were found to support the replication of *Leishmania infantum* and *Leishmania major* in their midgut for at least 3 days following an experimental infection [54,55]. Considering that the proposed Australian *Leishmania* vector is also a member of the family Ceratopogonidae, it is not unreasonable to suggest that this native midge may also be capable of transiently supporting the growth of clinically important *Leishmania* sp. With cases of imported human leishmaniasis becoming a regular occurrence in Australia, it is possible that the native midge vector will come into contact with human infecting *Leishmania* species, providing an opportunity for the establishment of a local transmission cycle. While the support for such an event is limited, it is not unprecedented and certainly warrants continued investigation.

## Conclusion

This represents the largest study to examine the molecular epidemiology of imported leishmaniasis in Australia. Of 206 patients suspected of harbouring a *Leishmania* infection over a six-year period, 55 were confirmed by PCR. The majority of infected patients had travelled to or immigrated from Afghanistan and *L*. *tropica* was the most common species identified. These results indicate that imported cases of leishmaniasis are an ongoing occurrence in Australia and highlights the need for Australian clinicians to consider leishmaniasis when assessing patients with a history of travel to or residence in endemic regions presenting with *Leishmania-*associated clinical manifestations.

## Supporting Information

S1 Table Published series of travel related leishmaniasis (DOCX)
