# Supplementary material for: Molecular Epidemiology of Imported Cases of Leishmaniasis in Australia from 2008 to 2014
Source: PLoS One. 2015 Mar 3;10(3):e0119212. doi: 10.1371/journal.pone.0119212 (PMC4348169; doi:10.1371/journal.pone.0119212)
Supplement: S1 Table — (DOCX) [file pone.0119212.s001.docx]

**S1 Supplementary Table.** Published series of travel related leishmaniasis

| **Country** | **Time period** | **# of cases** | **Region acquired (most to least frequent)** | **Travel type** | **Age range** | **Male sex** | **Disease (CL/ML [+MCL]/VL)** | **Speciation methodology (target)** | **% cases speciated** | **Species (most to least common)** |
| --- | --- | --- | --- | --- | --- | --- | --- | --- | --- | --- |
| Australia (current report) | 2008-2014 | 55 | Middle East, Americas, southern Europe, Asia | Tourists (51%), immigrants (38%), military (9%) | 1-76 | 75% | 52/0/3 | PCR-RFLP (ITS region) | 91% | *L. tropica, L. donovani, L. major, L. braziliensis, L. mexicana* |
| Australia [1] | 2005-2007 | 20 | South America, Middle East, Asia, southern Europe, Africa | Immigrants (55%), tourists (45%) | 8-59 | 85% | 18/0/2 | PCR-RFLP (ITS region ) | 100% | *L. tropica, L. braziliensis, L. infantum=L. mexicana, L. donovani* |
| Austria [2] | 2004-2010 | 14 | Americas, southern Europe = Middle East, Africa, Asia | NA | 1-76 | 71% | 14/0/0 | PCR + sequencing (ITS regions) | 43% | *L.donovani/ infantum = L.major^a^, L.guyanensis* |
| Europe^b^ [3] | 2000-2012 | 40 | Southern Europe | Tourists (83%) | 1-79 | 53% | 30/0/10 | PCR (NA) | 60% | *L. donovani/ infantum, L. tropica* |
| France [4] | 1999-2012 | 1154 | Americas, Africa | NA | NA | NA | 1051/NA/98 | PCR (NA) | 88%^c^ | *L. guyanensis, L. major, L. braziliensis, L. tropica* |
| France [5] | 2003-2005 | 35 | Africa | NA | 1-70 | 46% | 35/0/0 | Isoenzyme analysis, PCR (NA) | 49% | *L. major, L. infantum, L. tropica* |
| France [6] | 2006-2011 | 135 | Africa, Americas, southern Europe, Middle East, Asia | Tourists (86%) | 8mo-85 | 56% | 130^d^/5/0 | MLST (7 single copy coding DNA sequences) | 52% | *L. major, L. infantum/ chagasi, L. braziliensis, L. guyanensis, L. tropica, L. aethipoica, L. donovani= L. naiffi* |
| Germany [7] | 2000-2002 | 58 | Southern Europe, Americas, Middle East = Africa, Asia | Tourists (83%), immigrants (7%), workers (7%), visitors (3%) | 8mo-70 | 71% | 35 (CL+ML)/23 | PCR-RFLP (internal transcribed spacer (ITS) regions) | 57% | *L. donovanii, L. braziliensis, L. tropica, L. mexicana* |
| Germany [8] | 2000-2011 | 23 | Americas | NA | 17-69 | 70% | 23/0/0 | PCR-RFLP (ITS regions) | 100% | *L. braziliensis* |
| Israel [9] | 1998-2001 | 12 | South America | Tourists | 23-29 | 92% | 12/0/0 | L.braziliensis species specific PCR | 75% | *L. braziliensis* |
| Israel [10] | 2004-2005 | 7 | South America | Tourists | 21-24 | 71% | 7/0/0 | L.braziliensis species specific PCR | 100% | *L. braziliensis* |
| Italy [11] | 2001-2002 | 4 | Americas, Middle East = Africa | Tourists (75%), military (25%) | 23-63 | 100% | 3/1/0 | PCR-RFLP (repetitive genomic sequence) | 100% | *L. braziliensis, L. major = L. aethiopica/tropica* |
| Netherlands [12] | 2005 | 172 | Middle East (Afghanistan) | Military | NA | NA | 172/0/0 | PCR-RFLP (miniexon gene) | 40% | *L. major* |
| Netherlands [13] | 1990-2000 | 78 | Americas, Middle East, Africa, southern Europe, Asia | Tourists (most), military | 4-84 | 81% | 78/0/0 | PCR (NA) | 27% | *L. braziliensis, L. mexicana* |
| Netherlands [14] | 1998, 2004 | 39 | Americas (Belize) | Military | 18-48 | 100% | 39/0/0 | PCR + sequencing (18S rRNA gene) | 67% | *L. braziliensis, L .mexicana* |
| Netherlands [15] | 2005-2012 | 195 | Middle East, Americas, southern Europe, Africa, Asia | Military (52%), tourists, business, visitors | 2-78 | NA (most) | 185/2/8 | PCR + sequencing (mini-exon repeat) | 95% | *L. major, L. donovani/infantum, L. braziliensis/peruviana=L. guyanensis, L. tropica, L. panamensis, L. mexicana, L. naiffi* |
| Norway [16] | NA (2012^e^) | 6 (20)^f^ | Southern Europe, Asia, Americas | NA | 17-81 | 50% | 6/0/0 | PCR + sequencing (cytochrome b gene) | 15% | *L. infantum* |
| Spain [17] | 1995-2008 | 18 | Americas, Africa, Asia | Travellers (67%), immigrants (33%) | 17-55 | 72% | 12/4/2 | PCR (NA) | 39% | *L. braziliensis, L. major* |
| Switzerland [18] | 1999-2011 | 61 | Americas, Africa, Europe, Middle East | NA | 1-86 | 62% | 58/3/0 | PCR-RFLP (miniexon gene) | 100% | *L. major, L. infantum, L. braziliensis, L. panamensis=L. guyanensis, L. peruviana=L. tropica, L. aethiopica=L. donoviani=L. mexicana=L. naiffi=L. chagasi* |
| UK [19] | 2004 | 20 | Middle East (Afghanistan) | Military | 25 (mean) | 95% | 20/0/0 | PCR-RFLP (kinetoplast DNA) | 70% | *L. major* |
| UK [20] | 1997-1999 | 20 | Americas (most Belize) | Military (95%), work (5%) | 18-45 | 100% | 20/0/0 | L.braziliensis species specific PCR | 55% | *L. braziliensis* |
| UK[21] | 1997-2000 | 42 | South America, southern Europe, Asia, Africa | Tourists (45%), military (14%), immigrants-refugees (12%) scientific work (5%) | NA | 64% | 42/0/0 | species-specific PCR | 76% | *L. braziliensis, L. donovani, L. major, L. tropica* |
| UK [22] | 1998-1999 | 13 | Americas | Military | 19-35 | 100% | 13/0/0 | *L.braziliensis* species specific PCR | 100% | *L. braziliensis* |
| UK [23] | 1998-2009 | 223 | Americas, Middle East, southern Europe, Asia, Africa | Tourists (39%), military (24%), immigrants (15%), business | 2-86 | 69% | 223/0/0 | PCR-RFLP (ITS regions), PCR (kinetoplast DNA), species-specific PCR | 90% | *L.(Viannia) spp, L. mexicana, L. donovani, L. tropica=L. major, L. amazonensis, L. aethiopica* |
| USA [24] | 1990-1991 | 7 | Middle East (Saudi Arabia) | Military | 21-40 | 100% | 0/0/7 | Isoenzyme analysis | 71% | *L. tropica* |
| USA [25,26] | 2001-2006 | 1287 | Middle East (Afghanistan, Iraq, Kuwait) | Military | <30 (62%) | 96% | 1283/0/4 | Isoenzyme analysis | 24%^g^ | *L. major* |

1. Stark D, van Hal S, Lee R, Marriott D, Harkness J (2008) Leishmaniasis, an emerging imported infection: report of 20 cases from Australia. J Travel Med 15: 351-354.

2. Poeppl W, Oeser C, Grabmeier-Pfistershammer K, Walochnik J, Burgmann H (2013) Clinical findings and management of imported cutaneous leishmaniasis: report of 14 cases from Austria. Travel Med Infect Dis 11: 90-94.

3. Ehehalt U, Schunk M, Jensenius M, van Genderen PJ, Gkrania-Klotsas E, et al. (2014) Leishmaniasis acquired by travellers to endemic regions in Europe: A EuroTravNet multi-centre study. Travel Med Infect Dis 12: 167-172.

4. Lachaud L, Dedet JP, Marty P, Faraut F, Buffet P, et al. (2013) Surveillance of leishmaniases in France, 1999 to 2012. Euro Surveill 18: 20534.

5. Morizot G, Delgiudice P, Caumes E, Laffitte E, Marty P, et al. (2007) Healing of Old World cutaneous leishmaniasis in travelers treated with fluconazole: drug effect or spontaneous evolution? Am J Trop Med Hyg 76: 48-52.

6. Morizot G, Kendjo E, Mouri O, Thellier M, Perignon A, et al. (2013) Travelers with cutaneous leishmaniasis cured without systemic therapy. Clin Infect Dis 57: 370-380.

7. Harms G, Schonian G, Feldmeier H (2003) Leishmaniasis in Germany. Emerg Infect Dis 9: 872-875.

8. Harms G, Scherbaum H, Reiter-Owona I, Stich A, Richter J (2011) Treatment of imported New World cutaneous leishmaniasis in Germany. Int J Dermatol 50: 1336-1342.

9. Scope A, Trau H, Anders G, Barzilai A, Confino Y, et al. (2003) Experience with New World cutaneous leishmaniasis in travelers. J Am Acad Dermatol 49: 672-678.

10. Solomon M, Baum S, Barzilai A, Scope A, Trau H, et al. (2007) Liposomal amphotericin B in comparison to sodium stibogluconate for cutaneous infection due to Leishmania braziliensis. J Am Acad Dermatol 56: 612-616.

11. Antinori S, Gianelli E, Calattini S, Longhi E, Gramiccia M, et al. (2005) Cutaneous leishmaniasis: an increasing threat for travellers. Clin Microbiol Infect 11: 343-346.

12. van Thiel PP, Leenstra T, de Vries HJ, van der Sluis A, van Gool T, et al. (2010) Cutaneous leishmaniasis (Leishmania major infection) in Dutch troops deployed in northern Afghanistan: epidemiology, clinical aspects, and treatment. Am J Trop Med Hyg 83: 1295-1300.

13. Zeegelaar JE, Steketee WH, van Thiel PP, Wetsteyn JC, Kager PA, et al. (2005) Changing pattern of imported cutaneous leishmaniasis in the Netherlands. Clin Exp Dermatol 30: 1-5.

14. van Thiel PP, Zeegelaar JE, van Gool T, Faber WR, Kager PA (2011) Cutaneous leishmaniasis in three Dutch military cohorts following jungle training in Belize. Travel Med Infect Dis 9: 153-160.

15. Bart A, van Thiel PP, de Vries HJ, Hodiamont CJ, Van Gool T (2013) Imported leishmaniasis in the Netherlands from 2005 to 2012: epidemiology, diagnostic techniques and sequence-based species typing from 195 patients. Euro Surveill 18: 20544.

16. Blonski KM, Blodorn-Schlicht N, Falk TM, Faye RS, Clausen OP (2012) Increased detection of cutaneous leishmaniasis in Norway by use of polymerase chain reaction. APMIS 120: 591-596.

17. Perez-Ayala A, Norman F, Perez-Molina JA, Herrero JM, Monge B, et al. (2009) Imported leishmaniasis: a heterogeneous group of diseases. J Travel Med 16: 395-401.

18. Schonian G, Nasereddin A, Dinse N, Schweynoch C, Schallig HD, et al. (2003) PCR diagnosis and characterization of Leishmania in local and imported clinical samples. Diagn Microbiol Infect Dis 47: 349-358.

19. Bailey MS, Caddy AJ, McKinnon KA, Fogg LF, Roscoe M, et al. (2012) Outbreak of zoonotic cutaneous leishmaniasis with local dissemination in Balkh, Afghanistan. J R Army Med Corps 158: 225-228.

20. Hepburn NC, Tidman MJ, Hunter JA (1993) Cutaneous leishmaniasis in British troops from Belize. Br J Dermatol 128: 63-68.

21. Scarisbrick JJ, Chiodini PL, Watson J, Moody A, Armstrong M, et al. (2006) Clinical features and diagnosis of 42 travellers with cutaneous leishmaniasis. Travel Med Infect Dis 4: 14-21.

22. Seaton RA, Morrison J, Man I, Watson J, Nathwani D (1999) Out-patient parenteral antimicrobial therapy--a viable option for the management of cutaneous leishmaniasis. QJM 92: 659-667.

23. Wall EC, Watson J, Armstrong M, Chiodini PL, Lockwood DN (2012) Epidemiology of imported cutaneous leishmaniasis at the Hospital for Tropical Diseases, London, United Kingdom: use of polymerase chain reaction to identify the species. Am J Trop Med Hyg 86: 115-118.

24. Centers for Disease C (1992) Viscerotropic leishmaniasis in persons returning from Operation Desert Storm--1990-1991. MMWR Morb Mortal Wkly Rep 41: 131-134.

25. Center AFHS (2007) Leishmaniasis in relation to service in Iraq/Afghanistan, U.S. Armed Forces, 2001 - 2006. Medical Surveillance Monthly Report (MSMR) 14: 2-5.

26. Weina PJ, Neafie RC, Wortmann G, Polhemus M, Aronson NE (2004) Old world leishmaniasis: an emerging infection among deployed US military and civilian workers. Clin Infect Dis 39: 1674-1680.
